# Blood Pressure and Risk of Cognitive Impairment: The Role of Vascular Disease in Neurodegeneration

**DOI:** 10.3390/brainsci11030385

**Published:** 2021-03-18

**Authors:** Mar Carmona-Abellan, Malwina Trzeciak, Miriam Recio Fernández, Beatriz Echeveste, Laura Imaz, Maria-Rosario Luquin, Mario Riverol

**Affiliations:** 1 Department of Neurology, University Clinic of Navarra, 31008 Pamplona, Spain; mtrzeciak@unav.es (M.T.); mreciofernandez@gmail.com (M.R.F.); becheveste@unav.es (B.E.); limaz@unav.es (L.I.); rluquin@unav.es (M.-R.L.); mriverol@unav.es (M.R.); 2 Neurodegenerative Diseases Division, Health Research Institute Biocruces, 48903 Bizkaia, Spain; 3 IdiSNA, Navarra Institute for Health Research, 31008 Pamplona, Spain

**Keywords:** blood pressure, heart rate, cognitive impairment, dementia, cardiac stress testing, risk factors

## Abstract

(1) Background: Both cerebral vascular disorders and cognitive decline increase in incidence with age. The role of cerebral vascular disease and hemodynamic changes in the development of cognitive deficits is controversial. The objective of this study was to assess the cardiovascular response during cardiac stress testing in neurologically asymptomatic individuals who developed cognitive impairment several years after previous cardiac stress testing. (2) Methods: This was a retrospective cohort study of patients who underwent cardiac stress testing between January 2001 and December 2010. Patients were followed up until May 2015, and we selected those who developed cognitive dysfunction including dementia, mild cognitive impairment, and subjective cognitive decline, after the stress test. Heart rate and blood pressure both at rest and at peak exercise, and the mean R-R interval at rest were recorded. For each patient who developed cognitive impairment, we selected one matched control who did not show cognitive decline by the end of the follow-up period. (3) Results: From the cohort of 7224 patients, 371 developed cognitive impairment; of these, 186 (124 men) met the inclusion criteria, and 186 of the other patients were selected as matched controls. During follow-up, cognitive impairment appeared 6.2 ± 4.7 years after the cardiac stress test. These patients who had subsequently developed cognitive impairment had significantly lower at-rest systolic, diastolic, and mean blood pressure than controls (*p* < 0.05). Further, compared with controls, their maximum heart rate was significantly higher at peak exercise. (4) Conclusion: The results from this study suggest that differences in cardiovascular response to stress might be present in individuals who develop cognitive decline. These findings challenge the possibility of assessing blood pressure and heart rate variability at rest and during cardiac stress as potential risk factors associated with cognitive impairment.

## 1. Introduction

Cognitive disorders are becoming one of the most prevalent health issues in the developing countries, and in recent years, major efforts have been focused on identifying risk factors involved in cognitive decline. Among them, impaired blood pressure control and cerebrovascular damage have been associated with an increased risk of developing dementia [1,2] as uncontrolled blood pressure may lead to vascular brain damage and consequently impairment of cognitive function. 

Specifically, several studies have reported a U-shaped relationship between blood pressure and cognitive function [1,3,4,5,6,7,8]. In fact, low basal blood pressure seems to be associated with appearance of dementia [1], mild cognitive impairment (MCI) [9], subjective cognitive decline [10] and poor cognitive performance on the MMSE (MiniMental State Examination) [11]. These findings can be explained by the relative parieto-temporal hypoperfusion created by low blood pressure. In this regard, individuals with hypotension also had worse cognitive performance [12], and worse cognitive function has been observed after standing [13]. This might be related to lower blood pressure, especially diastolic, with consequent transient fronto-parietal hypoperfusion during orthostatic challenge. It is widely accepted that cerebral hypoperfusion leads to functional oligoaemia, hypoxia, oxidative stress, synaptic dysfunction, and neuroinflammation. All these events cause disruption of the blood-brain barrier and provoke biochemical changes such as microvascular degeneration, increased deposition of basement membrane proteins and perivascular amyloid. Also secretion of multiple neurotoxic and inflammatory factors such as interleukin 1–6, tumour necrosis factor-alpha, and hypoxia-inducible factor 1, resulting in beta-amyloid deposits [14]. Interestingly, all these changes have also been reported in damaged myocardium [15]. The damage provoked by vascular changes may cause microangiopathy, macroangiopathy, cerebral hypoperfusion and consequently promoting or accelerating neurodegeneration [14]. This hypothesis is supported by single photon emission computed tomography and magnetic resonance imaging studies that have shown changes in brain perfusion in the amygdala, insular cortex, anterior cingulated cortex and hippocampus [16]. Individuals with high systolic blood pressure and pulse rate were found to obtain better MMSE scores [17]. On the other hand, midlife systolic hypertension induces arterial stiffness, poor compliance [18] and a progressive reduction in baroreceptor sensitivity [19] which have been associated with cerebral white matter disease [10] and elevated plasma levels of Amiloid-β40 [3]. In this regard, higher blood pressure has been described a protective factor for cognitive functioning in the elderly [20]. Therefore, both hypertension, as a risk factor for cerebrovascular damage, and also low late-life diastolic blood pressure may be recognised as risk factors for the development of dementia [21]. These findings suggest the possibility of controlling a possible cause of brain damage to delay the cognitive decline appearance. 

In patients who develop dementia, cognitive impairment is preceded by 3 to 6 years by a reduction in blood pressure, especially systolic [22]. We hypothesized that some of these disturbances may be present under stress in the preclinical phase of cognitive decline, and that vascular instability precedes cognitive impairment. The aim of this study was to retrospectively assess cardiovascular response during cardiac stress testing (CST) in a cohort of neurologically asymptomatic individuals who developed cognitive impairment several years after the CST was performed. The results of this study might be helpful to identify individuals who are at risk of developing cognitive impairment and clarify whether the cardiac response to stress is present in this preclinical phase of cognitive decline. 

## 2. Patients and Methods

### 2.1. Participants

We conducted a single-center retrospective study of patients who underwent CST at the University Clinic of Navarra over a 10-year period (between January 2001 and December 2010). CST was indicated for patients who complained of chest pain or dyspnea or in the presence of vascular risk factors. After the CST, the participants were clinically followed up by their attending physicians until December 2015 and we identified those who had developed cognitive dysfunction. Patients diagnosed with subjective cognitive decline (SCD), MCI, or dementia were selected for this study provided that they met the following eligibility criteria: (a) adequate clinical information to allow follow-up of at least 24 months, and (b) diagnosis of cognitive impairment established by a neurologist applying the established criteria [23,24,25], including blood tests, brain magnetic resonance imaging (MRI) or computed tomography (CT), and neuropsychological tests.

Patients were excluded if they had (a) a diagnosis of cognitive impairment when the CST was performed; (b) cognitive impairment diagnosed on the same day as the CST (individuals referred to our Neurology Department for a check-up because they reported symptoms of memory loss); (c) a history of cardiac disease, including arrhythmias and coronary artery disease; or (d) depression or other psychiatric illnesses, obstructive sleep apnea, multiple sclerosis, or epilepsy. The rationale for the last set of exclusion criteria (d) was that previous research has indicated that cardiac autonomic tone as reflected by heart rate variability (HRV) is altered in patients with depression [26], obstructive sleep apnea, multiple sclerosis [27], and epilepsy [28]. Patients with clear autonomic dysfunction were excluded. Patients with coronary artery disease or a positive CST were also excluded from the analysis to avoid selection bias.

For each patient diagnosed with cognitive impairment, we selected a matched control from the CST cohort who had follow-up data and did not show cognitive decline by the end of the follow-up period. The remaining 1716 individuals potentially eligible as controls were different in age, prevalence of vascular risk factors, so they were not included as controls. Controls were individually matched for sex, age, body mass index, hypertension (yes/no), type 2 diabetes (yes/no), smoking habit (yes/no), dyslipidemia (yes/no), and antihypertensive drug use (yes/no), to control for confounding factors.

The study was conducted in accordance with the tenets of the Declaration of Helsinki.

### 2.2. Measures

CST was indicated for patients who complained of chest pain or dyspnea or who had relevant vascular risk factors (smoking, hypertension, type 2 diabetes, or dyslipidemia). The stress was induced by exercise on a treadmill according to the Bruce protocol [29] and the American Heart Association Statement for CST [30]. A Schiller Cardiovit CS-200 ergometer (Schiller AG, Baar, Switzerland) and an MTM 1500 treadmill machine (Schiller AG) were used for the exercise testing. The session started with 5 min of seated rest. At the end of this rest period, blood pressure (BP) was recorded from the right arm only. Electrocardiogram and heart rate (HR) were monitored continuously, and BP was measured during the last 30 s of each stage. The treadmill test was stopped when participants reached peak exercise. An active cool down was used, and vital signs were recorded every minute post-exercise for 5 min. The measurements obtained during the CST and subsequently analysed were systolic, diastolic and mean blood pressure at rest and at peak exercise, HR at rest and at peak exercise, percentage of the theoretical maximum HR, and increase in systolic and diastolic blood pressure after the exercise, as well as the mean R-R interval.

### 2.3. Statistical Analysis

Data collection and analyses were carried out between January and December 2017. Blood pressure and HR at rest and at peak exercise were assessed. Data are expressed as means ± standard deviations unless otherwise indicated. Characteristics of patients and controls were compared using a paired-samples t test. For comparisons of two or more means (e.g., HR or BP readings during the CST), an analysis of variance (ANOVA) was performed. The Tukey test was used to compare differences between each pair of means with appropriate adjustment for the multiple testing. In all cases, statistical significance was defined as *p* < 0.05. All analyses were performed using STATA version 14.

## 3. Results

### 3.1. Study Sample

**Figure 1:**
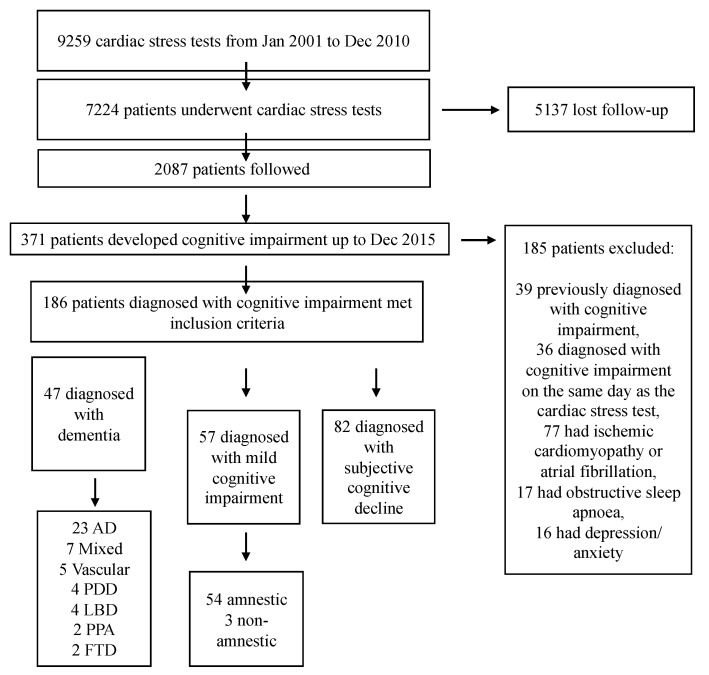
Flow diagram summarizing each stage of the study. AD, Alzheimer’s disease; FTD, frontotemporal dementia; LBD, Lewy bodies disease; PDD, Parkinson’s disease dementia; PPA.

In total, 9259 CSTs were performed between January 2000 and December 2010 at the University Clinic of Navarra on 7224 patients, with some patients undergoing several CSTs. From this cohort, 371 patients had developed cognitive impairment by December 2015, and of these, 186 met the inclusion criteria. Specifically, 47 had dementia, 57 MCI (54 amnestic MCI and 3 non-amnestic MCI), and 82 had subjective cognitive decline. Concerning the etiology of dementia, 23 patients met the diagnostic criteria for Alzheimer’s disease, while 7 had mixed dementia, 5 vascular dementia, 4 Parkinson’s disease/dementia complex, 4 dementia with Lewy bodies, 2 primary progressive aphasia, and 2 the behavioral variant of frontotemporal dementia. The flow diagram in Figure 1 summarizes the number of individuals at each stage of the study. Interestingly, seven patients with subjective cognitive decline and eight with MCI had progressed to dementia (due to Alzheimer’s disease) by the end of the follow-up period.

Most of the 186 patients were men, and most were overweight as indicated by the high mean body mass index; the overall mean age was 63 ± 9 years. At least 50% of the study sample had hypertension, dyslipidemia, and/or a smoking habit. The mean time from the CST to the diagnosis of cognitive impairment was 6.2 ± 4.7 years. As expected, there were no differences in age, sex, vascular risk factors, or treatment between patients and controls, as they were matched (*p* > 0.05). Characteristics of all participants at the time of CST are summarized in Table 1.

### 3.2. Comparisons of Cardiovascular Measures between Groups

Overall, patients who developed cognitive impairment had significantly lower values of systolic, diastolic, and mean blood pressure at rest than controls. Subgroup analysis showed that these differences compared to controls were also significant in patients with objective cognitive decline (both dementia and MCI groups), as well as in the patients with SCD. We did not find any differences between groups in basal HR. These data and *p* values are summarized in Table 2.

At peak exercise, there were no differences in blood pressure between patients who developed cognitive impairment and controls. Interestingly, the increase in systolic and diastolic blood pressure observed during exercise was larger in patients who developed cognitive impairment overall than in controls. In participants with SCD, only the increase in diastolic blood pressure during exercise was significantly larger than in controls (Table 2).

In addition, at peak exercise, patients who developed cognitive impairment had a significantly higher HR than controls. Again, subgroup analysis showed that compared to controls, these differences were also significant in patients with objective cognitive decline (both dementia and MCI) and patients with SCD (Table 2).

## 4. Discussion

We studied changes in blood pressure profile and cardiovascular response to cardiac stress in a group of patients with cognitive impairment to try to explain if changes in the response precede the appearance of cognitive decline. In this retrospective analysis of the CST performed on 7224 patients with no cognitive complaints, we found that 186 individuals developed cognitive impairment, including MCI, dementia or subjective cognitive decline cognitive after a follow-up period of 6 years.

Patients who developed cognitive impairment had significantly lower systolic, diastolic and mean blood pressure at rest than the control group, and these differences may not be explained by anti-hypertensive drugs or the presence of vascular risk factors, as patients and controls were matched for vascular risk factors, including antihypertensive drug use and presence of hypertension itself. Nevertheless, the analyses did not include number or class of antihypertensive drugs record, which is a limitation as beta-blocker use has been described to be associated with a lower risk of cognitive impairment compared with other antihypertensive drugs [31]. 

These groups of patients differed in basal blood pressure and, interestingly, after exercise, significant differences were found in maximal HR and increases in both systolic and diastolic blood pressure. At peak exercise, patients who subsequently developed cognitive dysfunction also had a lower basal blood pressure and after exercise there were no differences in maximum systolic or diastolic blood pressure. A greater increase in HR was observed, indicating that the baroreflex is intact in this group. The cardiovascular response to exercise is characterized by a greater increase in systolic and diastolic blood pressure from basal values and a larger increase in maximum HR in this cohort. Therefore, differences in cardiovascular profile are present in patients who develop cognitive deficits. Patients were followed-up over 5 years and neurological evaluation was performed in patients consulting for memory loss or referred by their physician when memory loss was suspected. This may lead to a selection bias, as some patients do not complain of memory loss or may develop cognitive impairment years after the study. In this regard, we selected controls participant from the cohort, with follow-up data confirming lack of cognitive decline over time. After follow-up most of the patients developed amnestic mild cognitive impairment, final diagnosis was associated with another type of cognitive impairment in some patients, which could add heterogeneity to the sample.

In this work, we investigated whether cardiovascular changes are present in patients who develop cognitive impairment, seeking to identify potentially modifiable risk factors and explain how cardiovascular response to exercise affect cognitive performance. Changes in cardiovascular function are linked to vascular rigidity, smoking and other vascular risk factors such as hypertension, all of them influenced by age. Over time, these changes may interact differently, and high blood pressure may become a risk factor for cognitive impairment in midlife, whereas it could be protective in late life, when low diastolic pressure is associated with cognitive impairment, due to brain hypoperfusion. High systolic blood pressure has been associated with risk of dementia in young elderly subjects, but not in older patients [32]. High blood pressure in midlife was linked with poorer cognitive functioning, however, this association declines with increasing age and in older people, there is a beneficial effect of high blood pressure on cognition [32]. In addition, with age, other chronic disease could be present, therefore, it would have been interesting to evaluate the presence of other chronic diseases in this cohort. Due to the retrospective design, these investigations were not performed. 

Our results are consistent with previous studies, describing patients with cognitive impairment and lower systolic and diastolic blood pressure than controls before developing clinical cognitive symptoms [10,22]. In this line, in older patients a decline in blood pressure has been observed around 3 years before the dementia is clinically evident [22]. We observed that resting baseline BP (both systolic and diastolic) were lower, but peak blood pressure was the same. Low blood pressure may reflect insufficient sympathetic response, due to the damage of the peripheral sympathetic nervous system, which mainly controls blood flow and arterial pressure, whereas the sympathetic cardiac innervation, involved in the control of the HR, is unaffected. In line with this hypothesis, hypotension and orthostatic intolerance have been also linked to dementia, through reductions in cerebral blood flow [33], cerebral hypoperfusion [10] and higher levels of white matter hyperintensities on neuroimaging. Impaired cerebral autoregulation in ageing contributes to this phenomenon, characterized by hypotension in late life [8] that may be present in patients with cognitive dysfunction. Therefore, chronic cerebral hypoperfusion and small vessel disease may contribute to cognitive impairment [34], and some of these changes may be present years earlier, and not necessarily associated with autonomic dysfunction. Interestingly, in SCD group of patients, an increase in diastolic blood pressure during exercise, larger than in controls, was measured, which may indicate that diastolic dysfunction could be one of the earliest changes. 

We observed lower BP, at least, a few years before cognitive decline, but with preserved capacity of the heart to increase the HR (intact baroreflex) during exercise. This is an interesting point, as compared with other neurodegenerative disorders such as Parkinson Disease (PD), we describe that there is still a compensatory response with the ability to increase the frequency, while in PD there is chronotropic insufficiency [35]. Nevertheless, the magnitude of the resting BP difference between groups is statistically significant but probably not clinically significant. We here address the possibility to measure changes in blood pressure and how these changes may be associated with cognitive impairment. In patients with vascular risk factors, hypertension may be present during midlife, causing continuous vascular damage and vessel rigidity, leading to ischemic lesions. Treating vascular risk factors including high blood pressure and optimising antihypertensive treatment seeking to avoid hypotension is recommended. 

In conclusion, the cardiovascular profile is altered before cognitive symptoms appear. In this retrospective cohort with long-term follow-up, differences in basal blood pressure, maximum HR and increases in systolic and diastolic blood pressure during exercise, were observed in patients who develop cognitive impairment in 6 years follow-up. Vascular risk factors and blood pressure should be measured and controlled to try to prevent the appearance of cognitive decline. 

## 5. Conclusions

The cardiovascular profile is altered before cognitive symptoms appear. In this retrospective cohort study with long-term follow-up, lower basal blood pressure with higher maximum HR and larger increases in systolic and diastolic blood pressure during exercise, compared with matched controls, were associated with cognitive impairment development in 6 years follow-up.

## Figures and Tables

**Table 1:** Sample characteristics at the time of cardiac stress testing overall and by final diagnosis.

Measures	Controls (*n* = 186)	CI after CST (*n* = 186)	Dementia after CST (*n* = 47)	MCI after CST (*n* = 57)	SCD after CST (*n* = 82)
Age, years	62.8 ± 9.3	62.8 ± 8.9	68.3 ± 6.5	64.7 ± 9.4	58.3 ± 7.6
Gender, men (%)	124 (66)	124 (66)	28 (59)	42 (73)	54 (65)
Body mass index, kg/m^2^	26.1 ± 7.3	26.9 ± 4.1	26.0 ± 3.9	27.8 ± 4.0	27.0 ± 4.0
Hypertension, yes (%)	106 (56)	106 (56)	32 (68)	33 (57)	41 (50)
Type 2 diabetes mellitus, yes (%)	59 (31)	59 (31)	20 (42)	23 (40)	16 (19)
Smoking, yes (%)	93 (50)	93 (50)	20 (42)	34 (59)	39 (47)
Dyslipidemia, yes (%)	132 (70)	132 (70)	33 (70)	45 (78)	54 (65)

Continuous variables are represented as mean ± standard deviation. CI, cognitive impairment; CST, cardiac stress testing; MCI, mild cognitive impairment; SCD, subjective cognitive decline.

**Table 2:** Cardiac stress test and heart rate variability measures overall and by subgroups.

	Controls (*n* = 186)	CI after CST (*n* = 186)	*p* Value ^a^	95% Confidence Interval	Controls (*n* = 104)	Dementia + MCI after CST (*n* = 104)	*p* Value ^a^	95% Confidence Interval	Controls (*n* = 82)	SCD after CST (*n* = 82)	*p* Value ^a^	95% Confidence Interval
Basal HR, bpm	78.0 ± 13.7	78.6 ± 13.7	0.703	−3.1; 2.1	78.2 ± 14.3	78.3 ± 13.1	0.952	−3.9; 3.7	77.8 ± 12.9	78.9 ± 12.8	0.575	−4.6; 2.5
Basal SBP, mmHg	128.9 ± 19.5	123.6 ± 18.5	0.001	2.1; 8.5	131.2 ± 19.2	126.2 ± 19.7	0.032	0.4; 9.6	126.0 ± 19.7	120.2 ± 16.5	0.014	1.2; 10.2
Basal DBP, mmHg	78.5 ± 11.3	75.5 ± 9.8	0.002	1.1; 4.8	78.3 ± 10.5	75.1 ± 10.4	0.016	0.6; 5.7	78.7 ± 12.3	76.0 ± 9.1	0.046	0.1; 5.4
Basal MBP, mmHg	94.7 ± 14.0	91.5 ± 11.7	0.006	0.9; 5.4	95.7 ± 13.3	92.1 ± 12.3	0.024	0.4; 6.7	93.4 ± 14.8	90.8 ± 10.8	0.018	−0.5; 5.8
Max HR, bpm	137.1 ± 30.2	144.6 ± 26.2	0.002	12.3; −2.7	131.4 ± 30.2	138.5 ± 28.3	0.038	−13.8; −0.4	144.3 ± 28.7	152.4 ± 21.0	0.024	−15.1; −1.1
Max SBP, mmHg	177.0 ± 32.7	179.2 ± 30.7	0.479	−8.1; 3.8	174.7 ± 34.0	175.9 ± 30.7	0.768	−9.2; 6.8	180.0 ± 30.9	183.4 ± 30.4	0.466	−12.4; 5.7
Max DBP, mmHg	87.4 ± 15.6	88.2 ± 12.5	0.585	−3.5; 1.9	86.5 ± 15.6	86.6 ± 13.1	0.953	−4.0; 3.7	88.6 ± 15.6	90.2 ± 11.5	0.423	−5.5; 2.3
Max MBP, mmHg	117.4 ± 19.5	118.6 ± 16.5	0.824	−4.6; 2.3	116.5 ± 20.2	116.2 ± 16.5	0.890	−4.4; 5.1	118.6 ± 18.6	121.6 ± 16.2	0.260	−8.3; 2.2
Δ SBP, mmHg	48.3 ± 32.9	55.0 ± 30.5	0.029	−12.6; −0.7	43.6 ± 34.6	49.5 ± 32.5	0.150	−14.1; 2.1	54.3 ± 29.7	62.0 ± 26.5	0.096	−16.4; 1.3
Δ DBP, mmHg	9.3 ± 13.1	12.7 ± 10.1	0.005	−5.7; −1	8.9 ± 14.8	11.6 ± 10.8	0.130	−6.3; 0.8	9.9 ± 10.5	14.1 ± 9.1	0.005	−7.1; −1.3
Mean R-R interval, ms	876.9 ± 159.4	909.7 ± 168.0	0.058	−66.7; 1.9	862.6 ± 150.4	904.8 ± 179.1	0.081	−89.6; 5.2	895.2 ± 169.4	916.1 ± 153.8	0.396	−69.7; 27.9

Continuous data are reported as mean ± standard deviation. ^a^ Paired samples *t*-test. Bpm, beats per minute; CI, cognitive impairment; CST, cardiac stress testing; DBP, diastolic blood pressure; HR, heart rate; MBP, mean blood pressure; MCI, mild cognitive impairment; MHR, maximum heart rate; SBP, systolic blood pressure; SCD, subjective cognitive decline.

## Data Availability

The data that support the findings of this study are available from the corresponding author, upon reasonable request.

## Standard Protocol Approval

This study was approved by the Institutional Review Board of the University of Navarra. Patients were informed that their data may be used for scientific purposes and provided their informed consent.

## Abbreviations

ANOVA	Analysis of variance
BP	Blood pressure
CST	Cardiac stress testing
CT	Computed tomography
HR	Heart rate
HRV	Heart rate variability
MCI	Mild cognitive impairment
MMSE	MiniMental State Examination
MRI	Magnetic resonance imaging
PD	Parkinson disease
SCD	Subjective cognitive decline
